# An up-date on health-related quality of life in myasthenia gravis -results from population based cohorts

**DOI:** 10.1186/s12955-015-0298-1

**Published:** 2015-08-01

**Authors:** M. I. Boldingh, L. Dekker, A. H. Maniaol, C. Brunborg, A. F. Lipka, E. H. Niks, J. J. G. M. Verschuuren, C. M. E. Tallaksen

**Affiliations:** Department of Neurology, Oslo University Hospital, Ullevål and Rikshospitalet, Ullevål, Pb. 4950 Nydalen, 0424 Oslo, Norway; Department of Neurology, Leiden University Medical Center, Leiden, The Netherlands; Department of Epidemiology and Biostatistics, Oslo University Hospital, Ullevål, Oslo, Norway; Institute of Clinical Medicine, University of Oslo, Oslo, Norway

**Keywords:** Health-related quality of life, SF-36, Myasthenia gravis, MuSK, Seronegative MG, Acetylcholine receptor, Thymoma, Population based, Burden of disease

## Abstract

**Abstract:**

Current available therapies control Myasthenia gravis (MG) reasonably well, but Health Related Quality of life (HRQOL) remains lower than expected. The aim was provide insights in how HRQOL in MG stands across borders and time, compare the scores to general population controls and other chronic disorders and assess the impact of potential predictors for quality of life such as a) clinical characteristics b) antibodies c) thymoma and d) treatment in a population-based cohort.

**Methods:**

We designed a population-based cross-sectional study including 858 patients, 373 from Norway and 485 from the Netherlands. The Short Form Health Survey 36 (SF-36) and a cross-cultural validated questionnaire were used. Data were in addition compared to the general population, other chronic diseases and previous studies.

**Results:**

Mean physical composite score was 59.4 and mental composite score 69.0 with no differences between the countries. The mean HRQOL score was lower in patients with bulbar and generalized symptoms (p < 0.001) compared to sex and age adjusted healthy controls, but not in patients with ocular symptoms or patients in remission. Multivariate analysis revealed that female gender, generalized symptoms and use of secondary immunosuppressive drugs at the time of testing were risk factors for reduced HRQOL.

**Conclusions:**

Remission and absence of generalized symptoms were favorable factors for HRQOL in MG patients. Historically, the HRQOL levels have not changed since 2001 and no new clinical predictors could be detected in this exhaustive population-based study. Further studies should explore the impact of non clinical factors like ethnic variations, socio-economic and hormonal factors on HRQOL.

**Electronic supplementary material:**

The online version of this article (doi:10.1186/s12955-015-0298-1) contains supplementary material, which is available to authorized users.

## Background

Myasthenia gravis (MG) is a heterogeneous neuromuscular autoimmune disease. Clinically, the symptoms range from mild ocular symptoms to severe generalized muscle weakness and disability. In the most severe cases the respiratory muscles are affected, causing problems breathing, a so-called “myasthenic crisis”. Nowadays, with optimal treatment, mortality is rare and most patients live normal lives. Nevertheless, health related quality of life (HRQOL) remains reduced compared to healthy controls in several studies (Table 1) [1–10].

**Table 1:** Overview over previous SF-36 studies amongst Myasthenia gravis patients

Author, country year	Number of patients	Design	Instrument	Objective	Outcome compared to norm population
Paul et al., USA, 2001 [2]	27	Cohort from Patient organization MGFA	SF-36	To describe HRQOL in MG patients compared to normative data	All, except mental health and bodily pain reduced compared to normative US population. Ratings of mood scale within 1 SD with US norm.
		Generalized 100 %			
Padua et al., Italy, 2002 [1]	46	Clinical cohort	SF-36	To evaluate the correlation of physician measures like Osserman and repetitive nerve stimulation to HRQOL outcomes	All domains largely reduced compared to normative Italian population.
		Remission: 6.5 %			
		Ocular: 4.3 %			
		Generalized: 89 %			
Rostedt et al., Sweden, 2005-2006 [5, 6] (3 publications)	42-48	Clinical cohort	SF-36 and MGQ validation	To correlate MGQ, SF-36 and degree of neuromuscular abnormalities measured by single fiber-EMG and repetitive nerve stimulation (RNS)	
		Remission: 20 %			
			SF-36 and MGQ versus SF-EMG		
		Ocular: 20 %			
		Generalized: 60 %			
Leonardi et al., Italy, 2010 [4]	102	Clinical cohort	SF-36, WHO-das II	To describe HRQOL and disability profiles according to ICF’s biopsychosocial model.	In patients without symptoms similar to general Italian population, greater difference with more symptoms.
Raggi et al., Italy, 2010 [3] (2 publications)		Remission: 24.5 %			
				To verify concordance between disease’s severity, HRQOL and disability in MG.	
		Ocular: 28.4 %			
		Generalized: 47 %			
Winter et al., Germany, 2010 [7]	37	Multicenter cohort	SF-36, EuroQoL, EQ-5D-index score	To compare HRQOL in patients with amyotrophic lateral sclerosis (ALS), fascial scapula humeral muscular dystrophy (FSHD) and Myasthenia Gravis.	All domains reduced, except bodily pain compared to normative German population
		Remission: 0 %			
			Comparison between ALS, MG and fascial scapula humeral muscular dystrophy.		
		Ocular: 45.9 %			
		Generalized: 43.2 %			
Twork et al., Germany, 2010 [9]	1518	Cohort from German Myasthenia Association^a^	SF-36	To analyze quality of life and life circumstances	More than one SD from normative population data Germany on the following domains:
					Female: PF, GH
					Male: PF, RP, GH, SF, RE
					Female > male
Kulkantrakorn, Thailand, 2010 [10, 29] (two publications),	71	Clinical cohort, two university hospitals^a^	SF-36	To study factors associated with QOL in MG patients	Females lower scores than males, however P-value is not given.
Basta et al., Serbia, 2012 [8]	230	Clinical cohort	SF-36, QMG, Hamilton rating, social support	To assess factors that might influence the HRQOL in MG patients	No population data available.
		Remission: 39.1 %			
		Ocular: 8.7 %			
		Generalized: 52.2 %			

Studies reporting data on only subgroups or validation studies were excluded

PF (Physical Functioning), RP (Role physical), BP (Bodily Pain), GH (General health), VT (Vitality), SF (Social Functioning), RE (Role Emotional), MH (Mental Health). Possible range 0-100; higher score indicates better functioning. MGFA classification (Myasthenia Gravis Foundation of America). Remission (MGFA 0), ocular (MGFA 1) and generalized (MGFA 2-4)^a^ clinical status not known. For information about the other scores or questionnaires we refer to original publications

These studies show that the disease has an extensive impact on physical, psychological and social wellbeing: the more severe muscle symptoms and disability, the lower the physical components of the outcome [1, 2, 7–10]. There are contradictory results as to what extend the mental wellbeing is affected in MG. Reported predictors for reduced HRQOL are among others use of prednisolone and side-effects, disease severity, depression, anxiety and disease duration, however, many others are also mentioned (Table 1). There are few larger studies and the impact of clinical subgroups based on antibodies, age of onset, thymus histology and clinical presentation remains unanswered [11–13].

Chronic immunomodulation with corticosteroid, other immunosuppressive drugs and thymectomy are often necessary to control the disease. Immunosuppressive medication produce marked improvement, however in many cases does the improvement not persist if treatment is discontinued. Consequently, treatment in MG is often a lifelong proces [14]. The choice of treatment depends on the severity of the symptoms, the specific subgroups and the pace of progression [14]. It is reported that MuSK MG and Thymoma MG are more likely to require secondary immunosuppressives than AChR MG and that elderly are more prone to side-effects [14–17]. As more advanced immunotherapy becomes available for MG patients, it is relevant to use HRQOL as an outcome for choice of treatment strategies. Long-term follow-up studies of representative MG cohorts assessing change of HRQOL during the last decades are lacking.

Up-to-date, there is no study reflecting the HRQOL of population-based MG cohorts covering the whole spectre of disease activity and immunological markers. Based on previous studies we hypothesized that serological subgroups, thymoma, disease course and treatment strategy are factors affecting HRQOL in MG patients. Our aim was to examine these factors and HRQOL in a MG cohort as large as possible, by combining population-based MG cohorts from two countries. We also compared the HRQOL outcomes of the MG patients to healthy controls, previous studies and other chronic diseases in order to get an impression of the burden of the disease.

## Methods

**Fig. 1:**
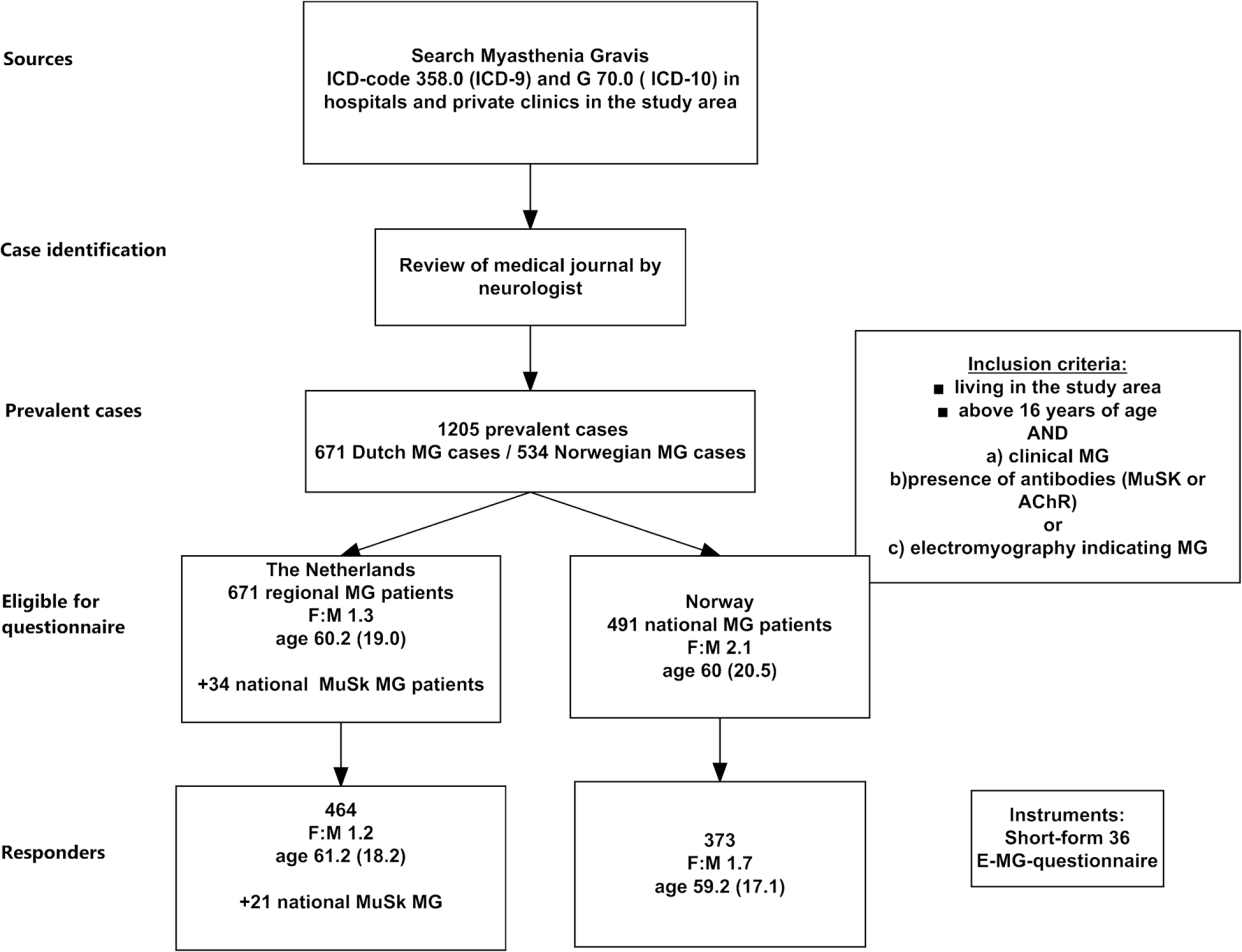
Overview over study procedure. The study was conducted among all MG patients in Norway and the contiguous regions of South- and North Holland in the Netherlands, including an additional MuSK Sample from entire Netherlands. The case identification and inclusion criteria were the same in both countries. The case search in the Norwegian study area was performed nationwide including 4 university clinics, 15 local clinics and 11 private clinics. Recruitment started: 01.01.2008. Recruitment stopped: 01.11.2009. The case search in the Dutch study was conducted in two regions and the affiliation to the geographical area was defined by postal code. The area had 4 university clinics, 25 local clinics and one private clinic. Recruitment started: 01.10.2011. Recruitment stopped: 01.01.2012. 43 patients were not eligible for questionnaire study because of change of address, dementia and other co-morbidities and delay in registration of ICD-code. Abbreviations: *ICD* = international classification diagnosis. *F*: *M* = female: male ratio

The design was cross sectional including two large population based MG cohorts from the entire Norway and a contigous province of South-Holland and North-Holland in The Netherlands (Fig. 1). In order to increase the sample size of MuSK MG in the serological subgroup analysis, we included a national population based sample (n = 34) from a prevalence study in the Netherlands [18].

### Study procedure

The details of the study are reported in a previous study [19, 20]. The patients were mailed a self-administered validated questionnaire including the Short Form-36 (SF-36) and questions about disease severity and current treatment for the last three months [21]. Reminders were sent to non-responders twice and missing data was completed by phone or mail.

Clinical characteristics, disease onset and course, presence of antibodies and thymus histology were collected from medical records.

### Definitions

Disease course was grouped into remission; either complete stable or pharmocological, ocular, bulbar or generalized symptoms at the time of testing. We subgrouped the ocular symptoms in those who started ocular and remained ocular throughout disease course (Ocular MG) and those who had residual ocular symptoms after generalized disease in the MG subgroup analysis. Non-steroid immunosuppresive medicines included azathioprine, cyclosporine, mycophenolate mofetil, tacrolimus, rituximab and cyclophosphamide.

### Instruments

The Norwegian and Dutch translations of SF-36 (version 1) was used to assess HRQOL [22, 23] in the past four weeks. This instrument is constructed for population surveys, is short and easy and has good psychometric qualities.

The SF-36 consist of 36 questions, organized into eight domains. The eight domains are physical functioning (PF), Role physical (problems with work or daily acitivites as a results of physical health, RP), Bodily pain (BP), general health evaluation (GH), Vitality (VT), Social functioning (SF), Role emotional (RE; severe problems with work or daily acitivites as a result of emotional health) and mental health (MH). All items are coded, scaled and transformed linearly from 0 (worst health) to 100 (best health). The first four can be summarized into a physical composite score (PCS; PF, RP, BP, GH) and the last four into a mental summary score (MCS; VT, SF, RE, MH).

### Control groups

We compared the two cohorts with published normative data, including reference data for chronic diseases (15 different conditions) and two population-based studies of Multiple sclerosis and Parkinsons disease [22, 24–26].

### Regulatory and ethical issues

We obtained informed constent from all participants and approval of the regional ethical committee of South-Norway, the ethical review committee of the Leiden University Medical Center (LUMC), The Netherlands, and the Medical Review Ethics Committee (METC) of South-West Holland.

### Statistical analysis

The statistical analysis was done using SPSS 22.0 (statistical package for social sciences, IBM, Armonk, NY, USA) and STATA 14 (StatCorp, Texas, USA). Scoring and calculation of the SF-36 scores were done according to the improved methods of Ware, McHorney and Sherbourne for version 1 [27, 28]. Method for impute missing data was followed for 0.7 % of the data. The MG patients were analyzed by domains and composite scores. Since the composite scores are considered as the most robust outcome measurement for the SF-36, we have focused on them in our comparisons. Differences in continuous variables were tested by independent sample t-tests and chi-square tests for contingency tables for categorical variables. When at least 25 % of expected cell frequencies were less than 5, Fischer’s exact test was applied. *P* < 0.05 was considered significant. Adjustment for the confounding effect of age, gender, disease severity and differences between both countries’ samples was done using linear regression analysis and stratum-specific estimates. We used the population-based cohort for all analysis (n = 837) either stratified by gender or country or pooled all together. For the immunological subgroup analysis, we included the responders of a national sample of MuSK MG patients (n = 21), leaving in total 856 MG patients for the analysis since the information about antibodies of eight patients was missing.

To examine the relationship between PCS/MCS and the significant covariates we used multiple regression analysis (p < 0.05) Afterwards, the model was employed on the MG subgroups with regard to antibodies to identify if the predictors played the same role in the subgroups.

### Results

858 MG patients (72 %) of the 1189 MG patients responded with a slightly higher response rate in Norway (76.2 %) than in the Netherlands (68.7 %). There was no difference considering age, sex, age onset, antibody profile and clinical remission rate between responders and non-responders of the questionnaire. The Dutch national sample of MuSK MG comprised more females (p = 0.011), but was otherwise similar to the Dutch regional MuSK MG cohort. No significant differences were found in the composite scores of HRQOL, and only minor differences in the subdomains (Table 2).

**Table 2:** Clinical characteristics of the population-based study cohort

	Total cohort, n = 837	Dutch MG Cohort, n = 464	Norwegian MG Cohort, n = 373	*P*-value	Adjusted *p*-value
Female [n;%]	491 (58.7)	256 (55.2)	235 (63.0)	0.022	0.749
Age [mean ± SD]	60.3 (17.6)	61.2 (18)^a^	59.2 (17.1)^a^	0.099	0.215
Married /cohabiting	572 (68.3)	316 (68.1)	256 (68.6)	0.881	0.870
Single/divorced/widow	265 (31.7)	148 (31.9)	117 (31.4)		
Mean age at onset [yrs ± SD]	45.8 (21.3)	49.1 (21.0)	41.8 (20.9)	<0.001	<0.001
Disease duration [yrs ± SD]	12.6 (12.2)	10.7 (11.0)	15 (13.2)	<0.001	<0.001
Antibody serology					
AChR MG ^b^	693 (82.2)	396 (85.3)	297 (79.6)	<0.001	<0.001
MuSK MG^b^	20 (2.4)	18 (3.9)	2 (0.5)		
SNMG^b^	115 (13.9)	44 (9.7)	71 (19)		
Missing n = 9 (0.9 %)					
Thymoma MG^b^	70 (21.2)	34 (25)	36 (18.8)	0.181	0.077
Age at onset [n;%]					
EOMG (<50 year)	397 (47.5)	196 (42.2)	202 (54.2)	<0.001	<0.001
LOMG (>50 year)	398 (47.8)	247 (53.2)	151 (47.9)		
Juvenile MG (<16 years)	41 (4.9)	21 (4.5)	20 (5.4)		
Disease course [n;%]					
Remission	196 (23.4)	97 (20.9)	99 (26.5)	<0.001	<0.001
Ocular	102 (12.2)	75 (16.2)	27 (7.2)		
Bulbar	90 (10.4)	44 (9.5)	46 (12.3)		
Generalized	449 (53.6)	248 (53.4)	201 (53.9)		
Current treatment [n;%]:					
Pyridostigmine	559 (66.8)	322 (69.4)	237 (63.5)	0.074	0.074
Prednisolone	279 (33.3)	157 (33.8)	122 (32.7)	0.731	0.966
Immunosuppressives	231 (27.6)	127 (27.4)	104 (28.0)	0.851	0.887
Combined	377 (45.0)	212 (45.7)	165 (44.0)	0.675	0.662
SF-36 [mean ± SD]					
Physical functioning	64.4 (29.8)	62.2 (30.7)	67.1 (28.3)	0.017	0.014
Role Physical	51.8 (43.7)	54.5 (44.0)	48.3 (43.2)	0.041	0.035
Bodily pain	69.3 (28.0)	72.6 (26.5)	65.1 (29.4)	<0.001	<0.001
General Health	52.4 (15.6)	52.7 (14.0)	52.4 (17.0)	0.802	0.896
Vitality	51.9 (22.7)	55.8 (21.3)	47.2 (23.4)	<0.001	<0.001
Social functioning	72.6 (26.9)	72.4 (26.4)	73.0 (27.6)	0.754	0.64
Role Emotional	75.7 (38.6)	79.5 (36.0)	71.1 (41.3)	0.002	0.002
Mental Health	75.1 (18.1)	73.4 (18.0)	77.6 (17.7)	0.001	0.001
Physical composite score	59.4 (23.6)	60.5 (23.0)	58.4 (24.3)	0.192	0.149
Mental composite score	69.0 (21.2)	70.3 (20.5)	67.5 (22.0)	0.053	0.064
Norm-based PCS	42.4 (11.0)	42.7 (11.1)	42.0 (10.9)	0.323	0.291
Norm-based MCS	50.5 (9.4)	50.6 (9.4)	50.5 (9.4)	0.905	0.902

Abbrevations: *MuSK MG* presence of Muscle specific tyrosine kinase antibodies, *AChR MG* presence of Acetylcholine receptor antibodies, *SNMG* no antibodies verified, *EOMG* early onset MG, *LOMG* late onset MG. Norm-based PCS/MCS (US norm 1998) with mean = 50 and SD =10

*P*-values are for difference between the Dutch and Norwegian MG cohort. Adjusted p-value for the potential confounding effect of differences in age and sex distribution between the cohorts

^a^ *p*-value < 0.005 between normative population data and study subjects

^b^information derived from medical charts

#### Comparison to healthy controls (Fig. 2)

**Fig. 2:**
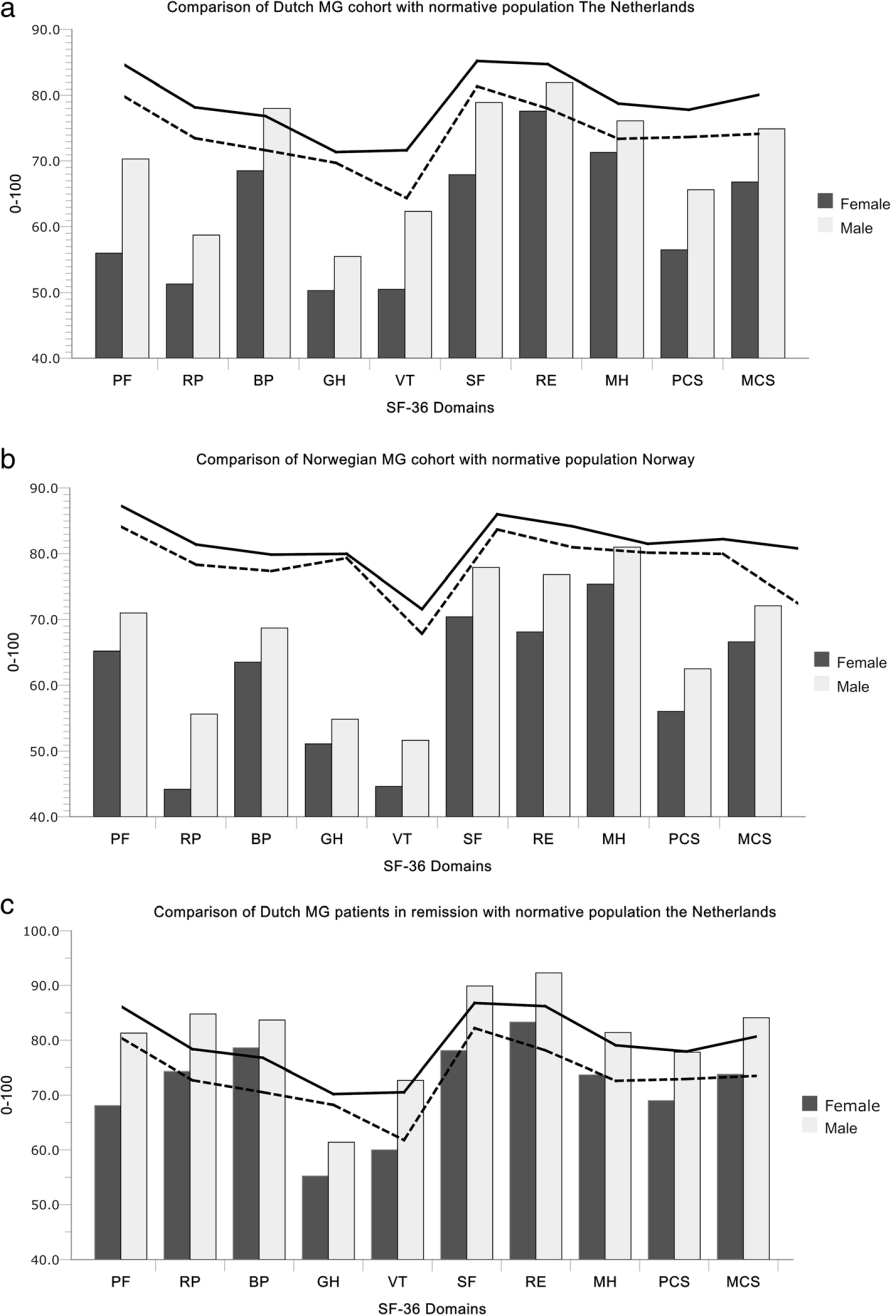
HRQOL in Myasthenia Gravis compared with healthy controls. The figure illustrates the population based MG cohorts in Netherlands (**a**) and the Norway (**b**) compared with healthy controls from their own countries. Healthy control data is provided by Loge et al.; Norway [24] and Aaronesen et al. [22]. In summary, MG patients scored lower than healthy controls and females scored lower than males and those in remission similar to healthy controls. The Score range from 0-100. Higher score indicate better Health related quality of life (HRQOL). Solid line is score of reference population for men, and dotted line is score of reference population for women in their respective countries. Horizontal axes show the 8 domains of SF-36 and composite scores

The total cohort of MG patients had lower scores than healthy controls (p-value <0.005, adjusted for age and gender), except for the domains of bodily pain (BP) and problems with daily activities due to mental health problems (RE). Female MG patients systematically scored lower than male MG patients, with significant p-values <0.001 on all domains and composite scores (Additional file 1: Table e-1).

Patients in remission (MGFA class 0) (n = 196, 23.4 % of the cohort) and those with ocular symptoms (MGFA class 1) (n = 102, 12.2 %) scored similar or better than their national normative controls on composite scores. For both groups, the HRQOL of the domains were similar to controls, except for the domain of general health (GH), were the score was significantly worse in male MG patients in both the Netherlands as in Norway.

MCS in particular showed age-dependent divergent results (Additional file 1: Table e-1). In the Netherlands, the MCS was lower for those below 60 years of age; 50 % of the females and 31 % of the males compared to controls. In contrast, the elderly MG patients from 70 years of age had reduced MCS in Norway compared to controls, representing 16.3 % of the females and 46 % of the males.

#### HRQOL in MG subgroups (Table 3, Fig. 3)

**Table 3:** HRQOL results in MG subgroups

	SNMG (n = 115)	AChR MG (n = 626)	MuSK MG (n = 41)^a^	Thymoma-MG (n = 70)	Adjusted *p*-value
Female [n, %]	63 (41.4)	376 (41.3)	27 (55.1)	37 (52.9)	NS
Age at study entry [yrs, SD]	54.2 (17)	61.7 (18)	52.2 (15)	61.5 (14)	*^b^
Mean age at onset [yrs, SD]	39.5 (18)	47.1 (22) ^c^	40.5 (17)	47.0 (17)^c^	NS
Mean age at diagnosis [yrs, SD]	41.8 (18)	48.7 (21) ^c^	43.1 (16)	50.4 (14.3)^c^	NS
Disease duration [yrs, SD]	13.5 (13)	13.4 (13)	11.8 (10)	10.2 (7.9)	NS
Disease course [n, %]					
Remission	24 (20.9)	151 (24.1)	11 (26.2)	16 (22.9)	NS
Ocular	20 (17.4)	76 (12.1)	1 (2.4)	5 (7.1)	*^d^
Bulbar	14 (12.2)	61 (9.7)	8 (19)	12 (17.1)	*^d^
Generalized	57 (49.6)	338 (54)	22 (52.4)	37 (52.9)	NS
Current treatment [n, %]					
Pyridostigmine	41 (35.7)	227 (36.3)	4 (9.5)	55 (78.6)	*^e^
Prednisolone	25 (21.7)	205 (32.7)	21 (50)	35 (50)	*^f^
Immunosuppressives	23 (20.2)	165 (26.6)	27 (65.7)	35 (50)	*^f^
Combined	37 (32.5)	274 (44.1)	34 (82.9)	46 (65.7)	*^g^
SF- 36 [mean, SD]					
Physical functioning	71.3 (26.3)	62.9 (30.3)	69.3 (26.7)	65.4 (27.6)	NS
Role Physical	53.8 (41.7)	51.1 (44.3)	49.4 (45.9)	55.6 (42.6)	NS
Bodily pain	68.9 (28.2)	69.7 (28.1)	73.3 (24.7)	68.4 (26.3)	NS
General health	52.8 (16.9)	52.6 (15.4)	51.2 (14.3)	51.4 (13.8)	NS
Vitality	48.9 (21.1)	52.2 (23.2)	54.8 (20.5)	52.0 (21.2)	NS
Social functioning	74.3 (26.5)	72.5 (26.9)	71.9 (27.3)	72.1 (27.6)	NS
Role Emotional	79.2 (35.6)	75.4 (38.8)	82.1 (35.0)	76.2 (39.0)	NS
Mental Health	74.5 (17.8)	75.6 (17.9)	69.3 (19.7)	73.7 (20.0)	NS
PCS	61.7 (22.5)	59.1 (23.9)	60.8 (22.6)	60.0 (22.2)	NS
MCS	69.2 (18.8)	69.0 (21.5)	69.5 (20.5)	68.5 (21.7)	NS
Norm-based PCS	43.6 (11.1)	42.1 (11.1)	43.7 (10.1)	42.6 (10.2)	NS
Norm-based MCS	50.2 (9.1)	50.7 (9.3)	49.2 (9.1)	49.9 (10.1)	NS

Adjusted *p*-value is calculated with age, sex, country and antibodies as dependent variables with logistic regression analysis

Footnote ^a^MuSk MG sample includes two MuSK MG patients from Norway, 19 MuSk patients from the study area North- and south Holland and in addition 21 from the rest of the Netherlands

*^b^SNMG and MuSK MG were younger than AChR MG and Thymoma MG

*^c^AChR MG and Thymoma MG patients were older than SNMG at onset and diagnosis

*^d^There were more ocular among the SNMG and more bulbar among thymoma MG and MuSK MG

*^e^MuSK MG patients used less pyridostigmine than AChR MG patients (p = 0.032)

*^f^MuSK MG patients used more prednisolone than AChR MG (p = 0.030) and SNMG (p = 0.017) and more secondary immunosuppressives than AChR MG (p = 0.006) and SNMG (p = 0.007)

*^g^Both MuSK MG and Thymoma MG used more combination therapy with secondary immunosuppressive drugs and prednisolone than SNMG and AChR MG

**Fig. 3:**
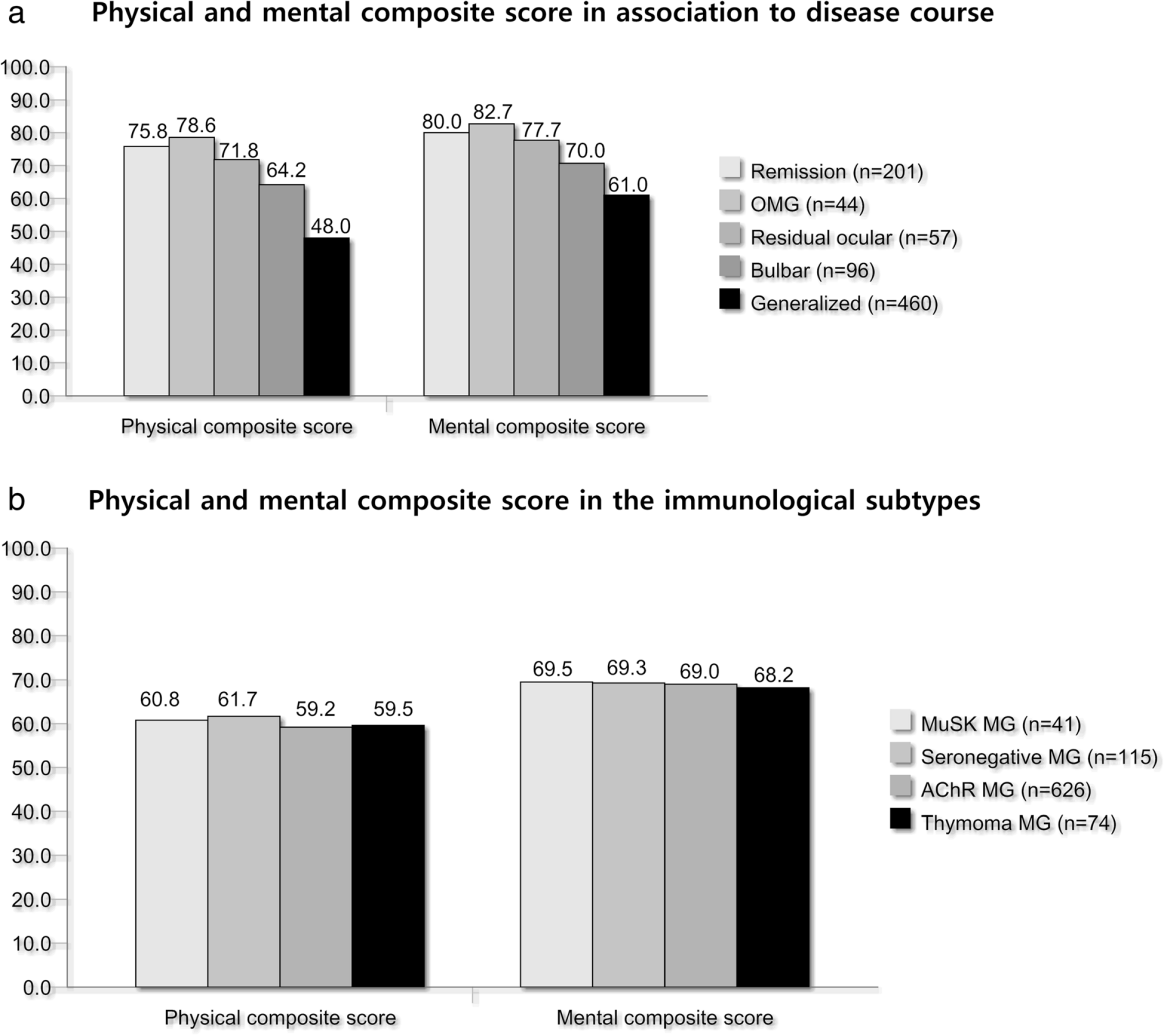
HRQOL by phenotype and antibodies. **a** MG patients in remission and absence of generalized symptoms scored significantly better than the other groups on both composite scores (p < 0.001). **b** Antibody profile did not affect HRQOL outcomes

Despite different disease course and more immuno-suppressives in MuSK MG and Thymoma MG, there were no differences in HRQOL. No differences were observed between early and late onset MG groups (adjusted for age). Patients with generalized and bulbar symptoms had lower HRQOL scores compared to those with OMG, residual ocular symptoms after generalized disease and those in remission (p < 0.001). No difference could be detected between those with OMG and those with residual ocular symptoms after generalized disease. No difference in PCS or MCS could be detected when stratifying the cohort into those early MG (0-3 years after onset) and longer follow-up (>3 years after onset).

#### HRQOL and treatment

Current treatment with non-steroid immunosuppressive drugs affected PCS negatively independent of disease activity (*p*-value <0.05 when adjusted for age, sex and disease activity). Prednisolone alone did not lower the PCS, but did so in combination with other immunosuppressive medicines (*p* = 0.002) . Thymectomy did not affect HRQOL, regardless of the histology results, thymoma, atrophy or hyperplasia.

#### Correlations between SF 36 composite scores and clinical variables

We used a multiple regression model with the PCS and MCS as outcome variables and gender, current disease symptoms and use of immunosuppresiva in general (prednisolon included) as possible predictors. Fourty-nine percent of the variance within Physical HRQOL were explained by these markers. Together the muscular weakness explained 44.2 % of the variance. Dyspnoe and muscular weakness in the legs were the strongest predictors (Table 4) compared to those without symptoms. The use of immunosuppressive drugs and gender were minor contributors each explaining around 4 %, *p* = 0 < 0.001.

**Table 4:** Regression coefficients of physical composite score in 850 MG patients

Predictor variable Physical composite score	Regression coefficient	95 % CI	*P*-value	Standard coefficient
R^2^	0.482			
Female gender	-4.69	-7.3, -2.4	<0.001	-0.102
Age	-0.30	-0.3, -0.2	<0.001	-0.225
Dyspnoe	-12.50	-15.3, -9.7	<0.001	-0.240
Muscular weakens in legs	-12.12	-15.2, -9.4	<0.001	-0.258
Muscular weakness in neck	-8.1	-11.7, -5.4	<0.001	-0.147
Muscular weakness in arms	-6.7	-10.3, -4.3	<0.001	-0.149
Dysphagia	-4.4	-8.4, -1.9	<0.001	-0.183
Use of immunosuppressive drugs	-4.6	-6.3, -1.7	<0.001	-0.088
**Model fit**	**R** ^**2**^	**R** ^**2**^ **change from basis model**		**Strongest predictors**
AChR MG	0.489	7 %	<0.001	Weakness legs and dyspnoe
MuSK MG	0.644	16.2 %	<0.001	Weakness legs and neck
SNMG	0.484	2 %	<0.001	Weakness legs and arms
Thymoma	0.588	10 %	<0.001	Weakness legs and dyspnoe

The same variables only explained 26 % of the Mental HRQOL in MG patients. Together dyspnoe and muscular weakness in legs, arms and neck contributed with 35.3 % of the variance. Gender was a minor marker, explaining around 5 %, and secondary immunosuppressivedrugs was not a significant risk factors for decreased mental health (Table 5).

**Table 5:** Regression coefficients of mental composite score in 850 MG patients

Predictor variable Mental composite score	Regression coefficient	95 % CI	*P*-value	Standard coefficient
R^2^	0.260			
Female gender	-4.8	-7.4, -2.2	<0.001	-0.111
Dyspnoe	-8.6	-11.6, -5.6	<0.001	-0.143
Muscular weakness in legs	-6.1	-9.2, -2.9	<0.001	-0.143
Muscular weakness in neck	-10.1	-13.4, -6.8	<0.001	-0.193
Muscular weakness in arms	-5.7	-8.9, -2.5	<0.001	-0.132
Age	-0.13	-0.2, -0.06	<0.001	-0.113
**Model fit**	**R** ^**2**^	**R** ^**2**^ **change from basis model**		**Strongest predictors**
AChR MG	0.271	11 %		Weakness neck
MuSK MG	0.271	11 %		Weakness neck, and female gender
SNMG	0.470	21 %		Weakness legs
Thymoma	0.455	19.5 %		Weakness neck

When applying the model on the different antibody subgroups, the model fit better for physical than for mental HRQOL and in particular better for MuSK MG and Thymoma MG (Tables 4 and 5).

#### Comparison to other chronic diseases (Fig. 4)

**Fig. 4:**
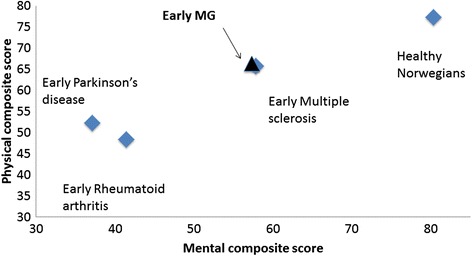
HRQOL in early MG patients compared to other chronic diseases. Early Norwegian Myasthenia Gravis patients from 0-3 years after onset scored similar to MS patients [25], but better than Parkinson’s patients [26] and Rheumatoid Arthritis patients with a similar disease duration [37]

We compared a stratified sample of Norwegian MG patients with symptoms in their early disease phase (0-3 years after onset) with patients with Norwegian Multiple sclerosis, Parkinson’s disease and Swedisch Rheumatoid Arthritis of similar disease duration and mean age. MG patients scored similar than MS patients and higher than Parkinson patients and Rheumatoid Arthritis patients (tested by *t*-test).

## Discussion

In this population-based double cohort study, we report a reduced self-perceived HRQOL in Myasthenia Gravis patients with bulbar and generalized symptoms, while patients in remission, those with OMG and those with residual symptoms after generalized disease scored similar to healthy controls. Generalized disease course, gender, and treatment with secondary immunosuppressives were risk factors for decreased HRQOL. Having a distinct subgroup with an antibody marker, thymoma or early or late onset subtype did not influence the HRQOL outcome.

**Fig. 5:**
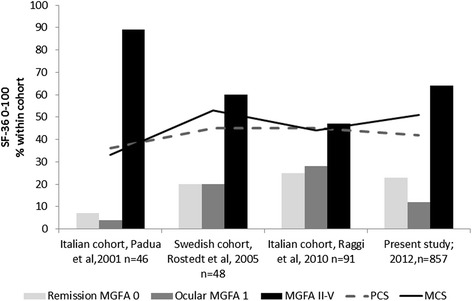
Overview over HRQOL measured by SF-36 from 2001-2012. Studies providing norm-based scoring are shown. Bars illustrate the distribution of MGFA score within the cohorts, lines illustrate the PCS and MCS. Vertical axis shows SF-36 score 0-100 for lines and distribution of MGFA class within the cohort. Padua et al. reported lower scores in 2002 than we did (p < 0.001), however the cohort consisted of fewer patients in remission (7 %) and 89 % in MGFA class II-IV [1]. Paul et al. 2001 [2], provided not norm-based scoring, but PCS 57.6 (27) and 65.5 (24.8) were not significant different from our study

The reduced quality of life was determined by lowered physical capacities, but psychological wellbeing was affected in roughly half of the patients as well. There was no differences in HRQOL between MG patients in Norway and the Netherlands. Pooling the data could therefore be done without affecting the validity of the results and confirms the reliability of the results. Put into an historical context, the HRQOL has not changed much for MG patients over the last 10-15 years and we found the same levels as reported in 2001 [2, 5–10] (Fig. 5). The reduced results of Padua et al. [1] may be explained by a low percentage in clinical remission and many patients with generalized symptoms compared to our cohort. The result of “the more generalized disease or active” disease, the poorer HRQOL” is in accordance with other reports [1, 3, 7, 8, 29]. Leonardi et al. [4] reported that patients in remission scored similar to Italian general population, supporting our findings from the Dutch and Norwegian MG patients. We did not find any harmful impact of antibodies or thymectomy in accordance with other studies [1, 8, 30]. Our findings of normal levels of HRQOL in ocular MG is supported by another study of 91 ocular Italian MG patients [11]. In contrast, a Japanese study reported QOL impairment of 123 ocular MG patients in those who not responded to therapy [13]. One explanation of these contradictory results may be patients selection, since ocular MG includes several symptoms (ptosis, diplopia, complete ophthalmoplegia), with potentially different grades of disability. Additionnally, the use of another questionnaire and cultural factors may account for the difference.

In addition to generalized weakness, dyspnoe and dysphagia, female gender, usage of secondary immunosuppressives were minor risk factors to reduced HRQOL, similarly to results form previous studies [9, 30, 31]. Some of the gender variability could be explained by a general gender effect as the pattern is similar as seen in the normal population [22, 24]. However, we know that hormonal factors affect the symptoms [32, 33] and could thus also contribute. The second finding may suggest that long term immunosuppressive therapy may not be as harmless as assumed. This is supported by a HRQOL study from Japan, which documented an impact of steroids and calcineurin inhibitors in two studies [30, 31]. The negative effects may be related to side-effects or to the burden of constant need of medication. As reviewed by Sanders [14], the initial goal of therapy should be reducing weakness as much and as quickly as possible. Balancing side effects of immunotherapeutic agents and treatment effect remains a challenge for the clinician. The correlation between secondary immunosupressive agents and reduction of HRQOL in MG patients indicates that it is important to keep in mind the benefits of usage contra the side effects, and aim at reducing dosage to lowest possible maintenance dose. This may in particular be important for ocular MG in which no RCT has yet documented effect of any secondary immunosuppressive treatment, neither on symptoms remission nor on prevention of generalized disease [34, 35].

Early MG scored similar to early MS, however when looking at longer follow- up reports HRQOL turns worse in MS [36]. The explanation is probably because MS and Parkinsons’ disease have often a more progressive course with no or less treatment that stops progression. Rheumatoid arthritis, on the other hand, is associated with more pain and disability than MG which can explain the marked lower scores. Improvement of HRQOL scores are reported in RA within the same patients, but also improvement over the last decades [37, 38]. According to Grob et al.’s lifetime study of MG, the distribution, severity and course of disease is determined within the first two years [39] and many MG patients experience the worst phase of the disease in these years. One should therefore expect improvement in HRQOL with longer disease duration. Our study design was cross-sectional and limits therefore interpretation of HRQOL over time in the same patient. Hence, follow-up studies both of individuals over time are needed to learn more about HRQOL in MG. With better treatment strategies and therapautic options we do also expect improvement over the last decade, however the comparison with earlier studies did not confirm this. Limitations in these inter-study comparisons are that the patients are not matched in the study and many variables are not controlled, therefore the results are purely indicative.

The main strength of the study is the design, which is a true population-based, cross sectional survey of two well defined groups. Finding the same levels of HRQOL in both countries makes the results more reliable. All clinical subtypes of MG were present in sufficient numbers to give useful results. Disease specific questionnaires have been advocated for MG, and indeed may enable a more specific follow-up for the patients [40]. Our intention was, however, to use a broadly validated questionnaire to provide insights in how HRQOL in MG stands across borders and time, compare the scores to general population controls and other chronic disorders. Correlations between the SF-36 and MG disease status has been proven in several studies, and may thus be trusted to give a fair picture of the burden of MG disease. A more disease specific instrument would be able to detect smaller changes in vision, speaking, chewing and swallowing better [1, 4–6], which was not the purpose in this study. Since the SF-36 has norm-based scoring, group level results of this study can be used to compare MG patients across countries and further improve the quality of health care systems in places where it is not optimal.

## Conclusion

Taken together, this population based study of 857 MG patients found the same HRQOL levels as in 2001 [2], indicating no improvement in quality of life for MG patients despite more available immunotherapy choices and better diagnostics of MG subgroups. Although we included a large sample and patients with different clinical characteristics, we could not find any new clinical predictors for reduced HRQOL compared to the other studies. Therefore we suggest that the impact of non-clinical factors like job, socio-economic factors, physical activity and hormonal factors on HRQOL in MG patients should be explored in order to improve the quality of life in MG patients.

## Additional file

Additional file 1:
**Table e-1.** Mean SF-36 scale scores of MG patients versus healthy controls (stratified by gender).
