# Supplementary material for: An up-date on health-related quality of life in myasthenia gravis -results from population based cohorts
Source: Health Qual Life Outcomes. 2015 Aug 1;13:115. doi: 10.1186/s12955-015-0298-1 (PMC4522107; doi:10.1186/s12955-015-0298-1)
Supplement: Additional file 1: — Table e-1. Mean SF-36 scale scores of MG patients versus healthy controls (stratified by gender). [file 12955_2015_298_MOESM1_ESM.docx]

Table e-1 Mean SF-36 scale scores of MG patients versus healthy controls (stratified by gender)

1a

|  | Dutch MG cohort, female  n=258 | General Dutch population Sample,  Female n=767 | P-value  (sex adjusted) | Dutch MG cohort, Male  n=209 | General Dutch population Sample,Male, n=976 | P-value  (sex adjusted) | Adjusted p-value for age¹ (Complete Dutch cohort)² |
| --- | --- | --- | --- | --- | --- | --- | --- |
| Physical Functioning | 55.8 (31.6) | 80.4 (24.2) | <0.001 | 70.3 (27.6) | 85.4 (21.0) | <0.001 | <0.05 |
| Role Physical | 51.3 (43.6) | 73.8 (38.5) | <0.001 | 58.7 (44.4) | 78.7 (34.1) | <0.001 | <0.05 |
| Bodily Pain | 68.5 (27.9) | 71.9 (23.8) | 0.027 | 78.0 (23.4) | 77.3 (22.7) | 0.5685 | NS |
| General Health | 50.3 (14.1) | 69.9 (20.6) | <0.001 | 55.5 (13.3) | 71.6 (20.6) | <0.001 | <0.05 |
| Vitality | 50.5 (19.9) | 64.3 (19.7) | <0.001 | 62.3 (21.4) | 71.9 (18.3) | <0.001 | <0.05 |
| Social Functioning | 67.9 (27.6) | 82.0 (23.5) | <0.001 | 78.9 (22.8) | 86.0 (21.1) | <0.001 | <0.05 |
| Role Emotional | 77.6 (37.5) | 78.5 (35.7) | 0.447 | 81.9 (33.8) | 85.5 (29.9) | 0.0881 | NS |
| Mental Health | 71.3 (18.3) | 73.7 (18.2) | 0.020 | 76.1 (17.5) | 79.3 (16.4) | 0.016 | *<60 years |
| Physical Composite Score | 56.5 (23.3) | 74.0 (26.8) | <0.001 | 65.6 (21.5) | 78.3 (29.8) | <0.001 | <0.05 |
| Mental Composite Score | 66.8 (20.5) | 74.5 (24.2) | <0.001 | 74.9 (19.7) | 80.7 (21.4) | <0.001 | *<60 years |

1b

|  | Norwegian MG Female cohort n=235 | Healthy Norwegian female Sample, N=1111-1184 | P-value | Adjusted p value by age and sex | Norwegian Male MG cohort, n=139 | Healthy Norwegian Male sample, n=1072-1113 | P-value | Adjusted p-value for age and sex. |
| --- | --- | --- | --- | --- | --- | --- | --- | --- |
| Physical Functioning | 65.2 (29.4) | 84.8 (20.8) | <0.001 | <0.05 | 71.0 (25.5) | 89.8 (15.5) | <0.001 | <0.05 |
| Role Physical | 44.2 (43.4) | 75.4 (37.7) | <0.001 | *<60 years | 55.6 (42.2) | 80.5 (33.6) | <0.001 | NS |
| Bodily Pain | 63.5 (30.3) | 73.0 (26.6) | 0.002 | NS | 68.7 (26.0) | 77.2 (25.0) | 0.027 | NS |
| General Health | 51.1 (17.6) | 76.3 (22.5) | <0.001 | <0.05 | 54.8 (15.9) | 77.4 (21.3) | 0.001 | <0.05 |
| Vitality | 44.6 (23.1) | 56.9 (21.2) | <0.001 | <0.05 | 51.6 (23.1) | 63.2 (19.9) | <0.001 | <0.05 |
| Social Functioning | 70.4 (27.6) | 83.7 (23.1) | <0.001 | <0.05 | 77.9 (26.5) | 87.6 (20.9) | <0.001 | *>60 years |
| Role Emotional | 68.1 (43.1) | 79.1 (34.6) | <0.001 | NS | 76.8 (37.0) | 84.5 (29.7) | 0.001 | NS |
| Mental Health | 75.4 (19.3) | 77.7 (17.0) | 0.107 | NS | 81.0 (14.9) | 80.0 (15.8) | 0.485 | NS |
| Physical Composite Score | 56.0 (24.8) | 77.4 (26.9) | <0.001 | <0.001 | 62.5 (22.9) | 81.2 (23.8) | <0.001 | *>70 years |
| Mental Composite Score | 66.6 (23.0) | 64.3 (23.9) | 0.803 | NS | 72.1 (19.0) | 78.8 (21.5) | <0.001 | *> 70 years |

Table legend:

Healthy controls were adapted from Loge et al; Scand J Soc Med, 1998 (Norway) and Aaronsen et al; J clin Epidemiol, 1998 (Netherlands).

Respondents SF-36 Norway: n =2323. Mean age 44.9 years (16.5), range 19-80; Gender distribution 51% female /49% male

Respondents SF-36 the Netherlands: n=1742 Mean age 47.6 (18.0), range 16-94. Gender distribution 54% female/46% male.

Abbreviations comment section: NS: not significant. * Significant above / below a particular age group
